# Supplementary figures and images for: TMEM174 Deficiency Reduces Longevity by Promoting Phosphate-Driven Vascular Calcification
Source: Res Sq. 2026 Jun 1:rs.3.rs-9857363. Preprint. [Version 1] doi: 10.21203/rs.3.rs-9857363/v1 (PMC13252570; doi:10.21203/rs.3.rs-9857363/v1)

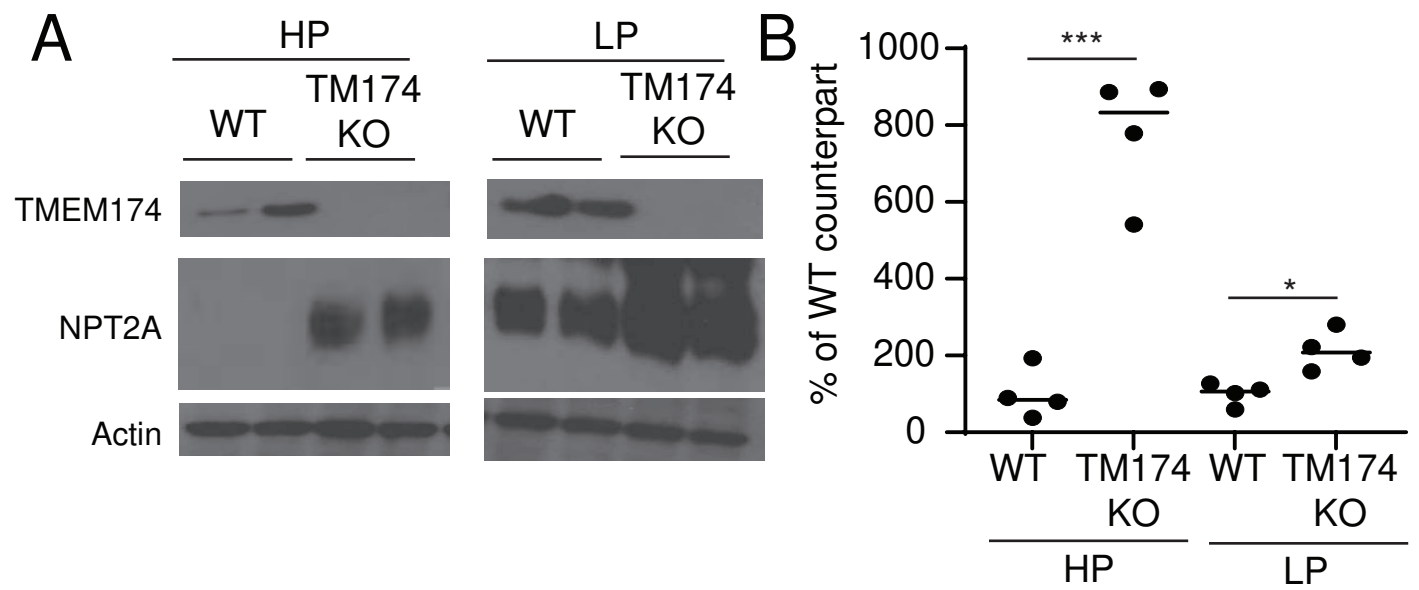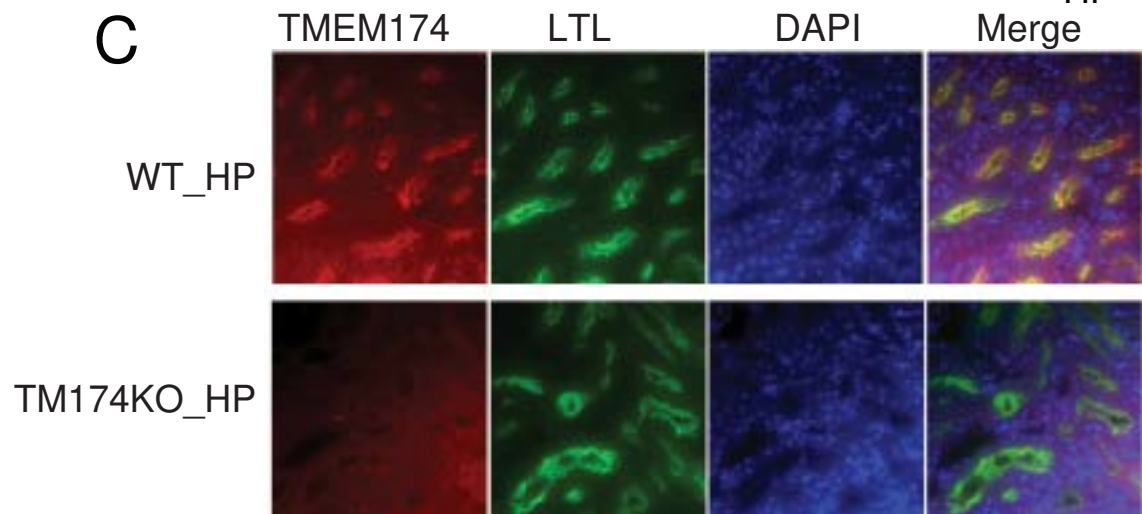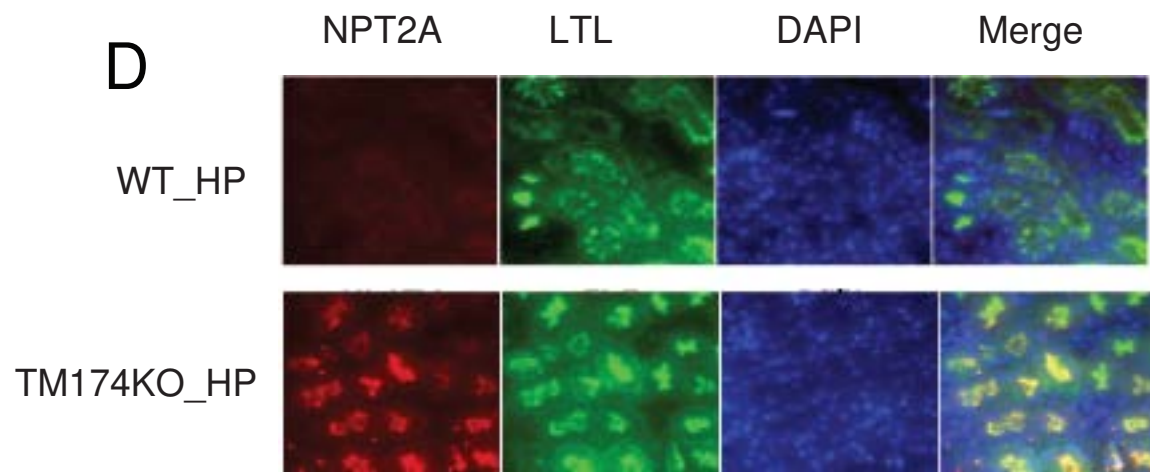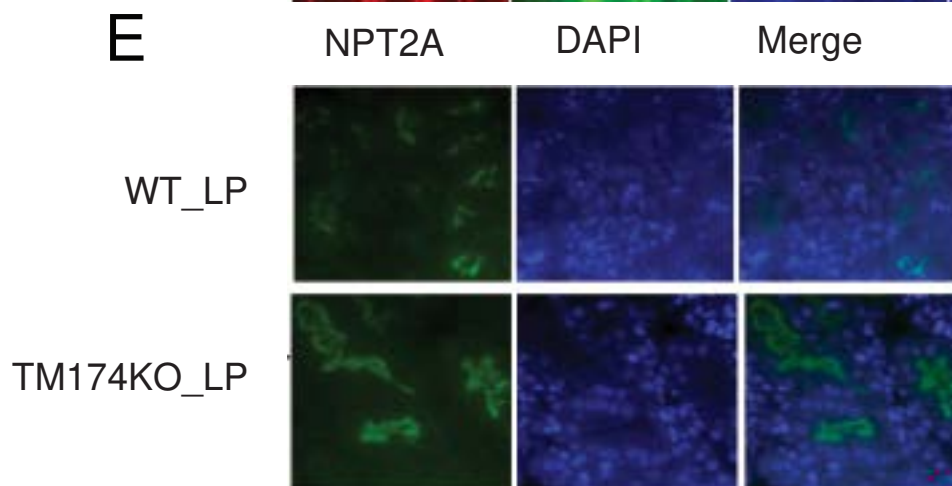

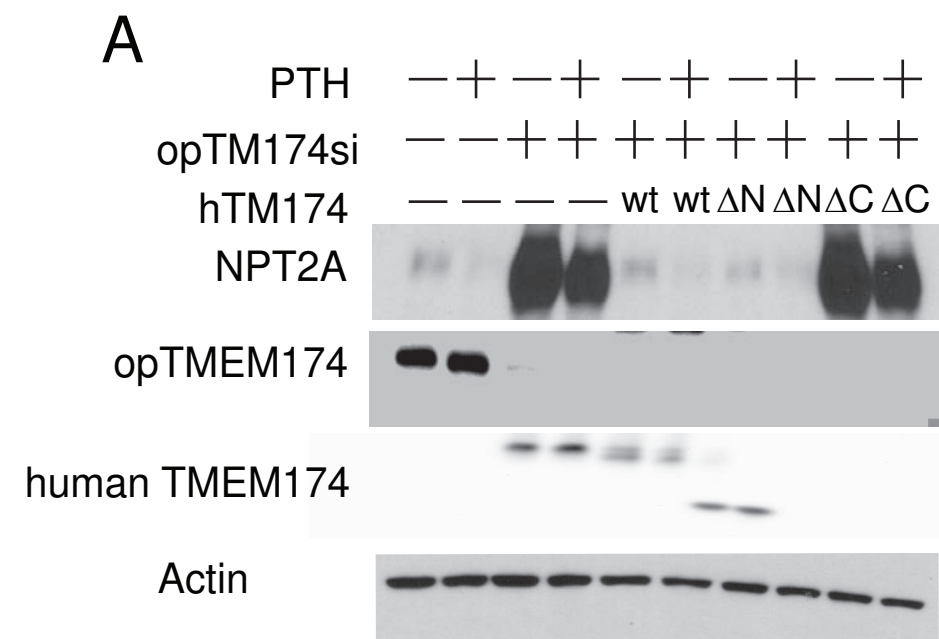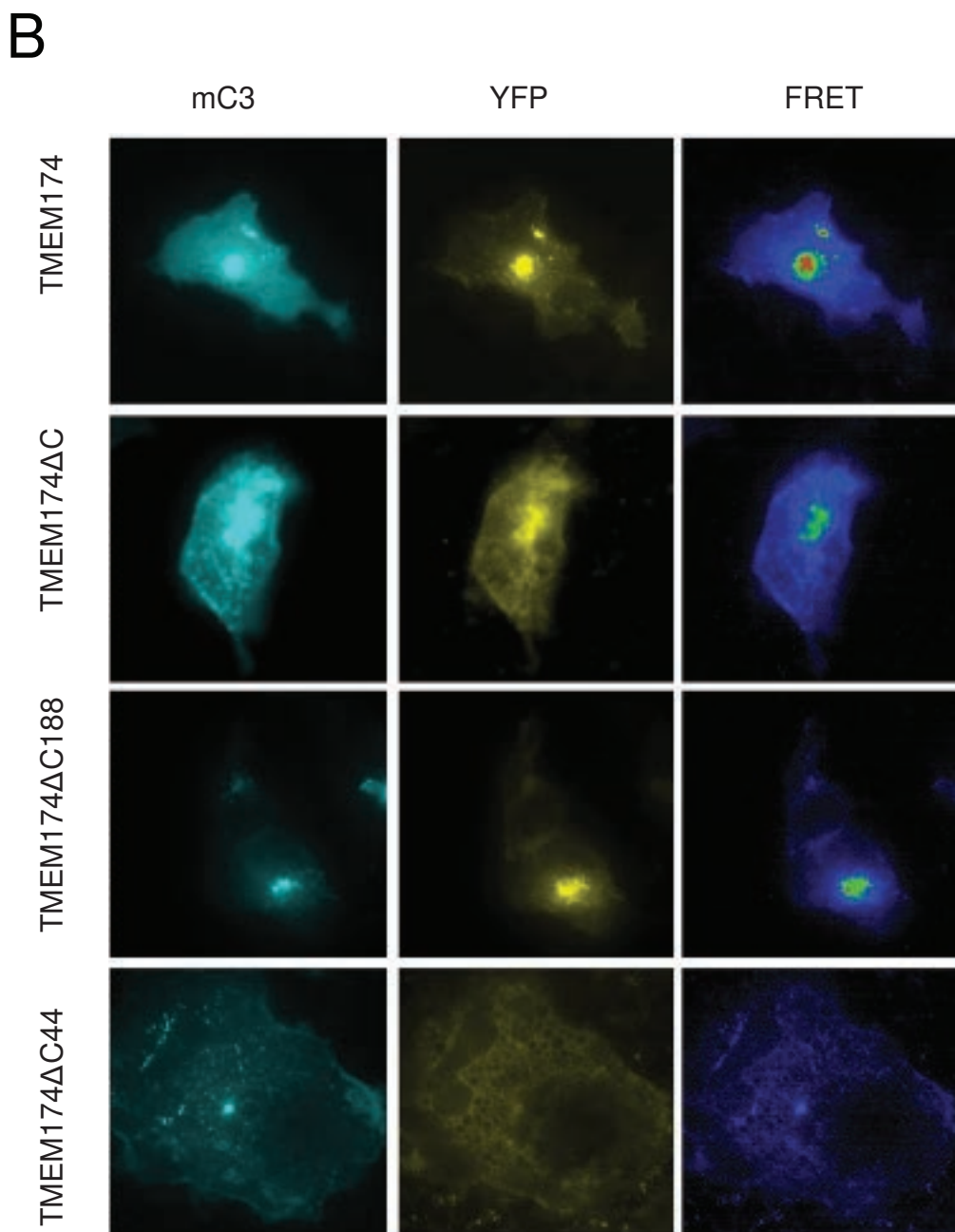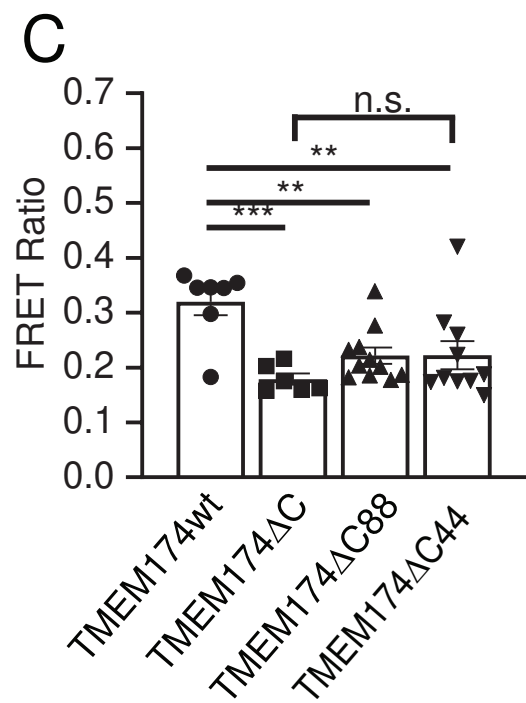

S. Figure. 2

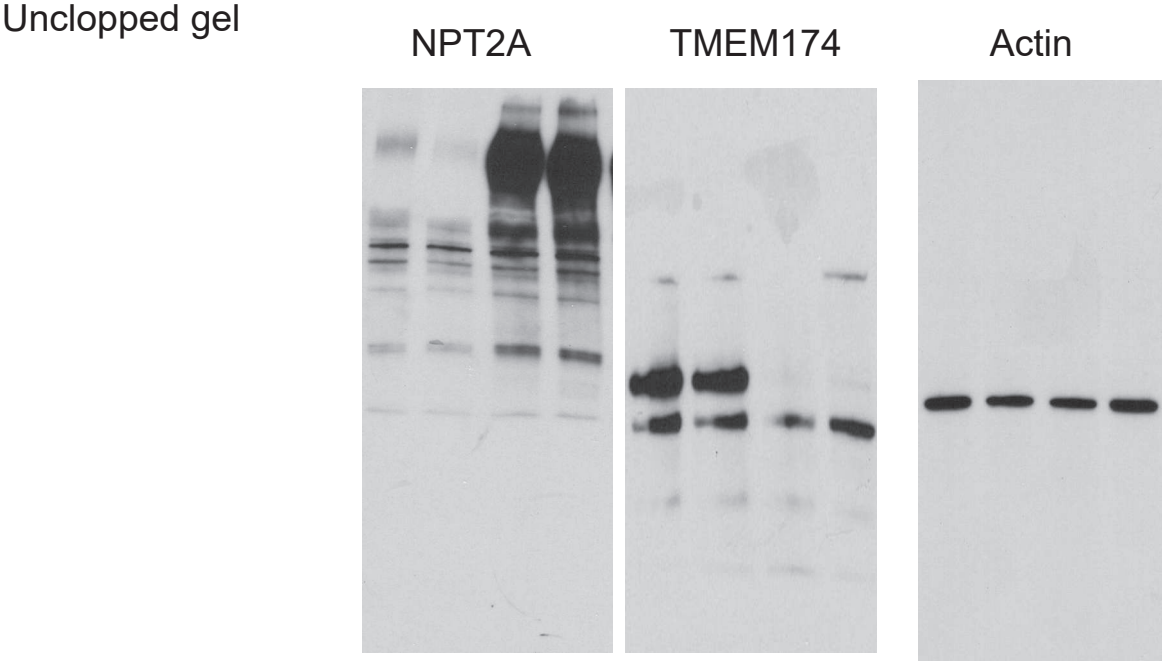

Fig. 5

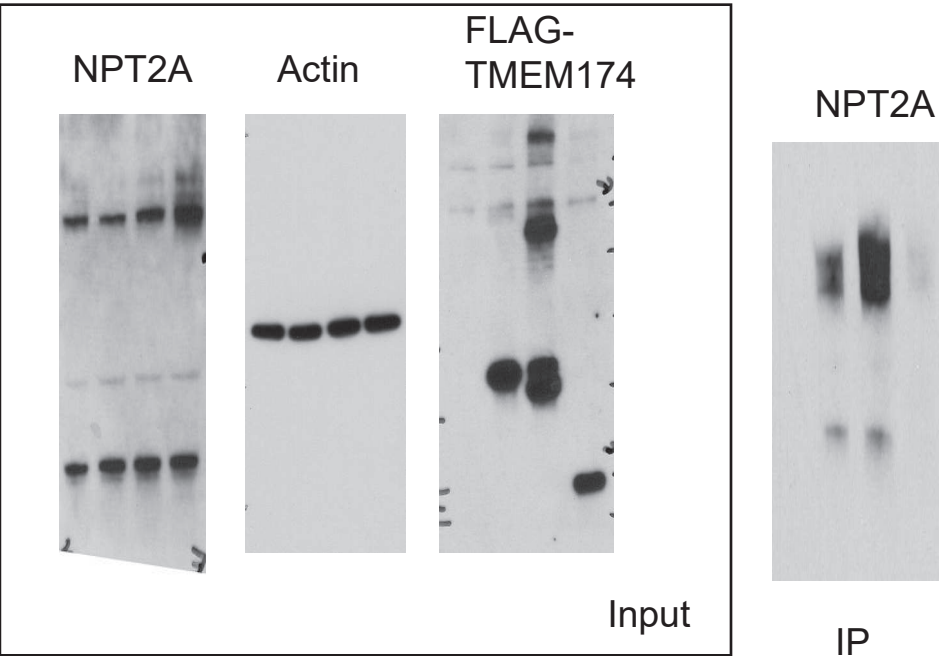

Supplement: 1 — Supplemental Figure 1. Low phosphate diet attenuates the effect of TMEM174 deficiency on NPT2A protein expression in the renal proximal tubules. A) Immunoblot and B) densitometry analyses of TMEM174 and NPT2A in the renal BBM from mice fed high phosphate and low phosphate diets. The renal BBM fraction was isolated using the Ca2+ precipitation method. Immunofluorescence analysis of C) TMEM174 and D) and E) NPT2A in TMEM174KO mice fed high-phosphate and low-phosphate diets. NPT2A and TMEM174 were detected with anti-mouse NPT2A and anti-mouse TMEM174 polyclonal antibodies coupled with LTL (a proximal tubule specific marker). Supplemental Figure 2. The TMEM174 C-terminal end is critical in the regulation of NPT2A. A) Immunoblot analysis of TMEM174 and NPT2A in TMEM174 knockdown OKP cells reconstituted with human TMEM174 mutants in the absence and presence of PTH. OKP cells were treated with opossum-specific TMEM174 for 7 days and then TMEM174 expression was reconstituted by human TMEM174 mutants. After two days at 100% confluency, cells were treated with 10nM PTH/0.1%BSA for 1 hour. Total cell lysate was isolated with RIPA buffer. Opossum and human TMEM174 were detected with anti-opossum TMEM174 specific antibody (Biomatik) and anti-FLAG monoclonal antibody (Sigma), respectively. B) The representative images for each set of experiments showing EYFP-NPT2A and TMEM174-mC3 wt and mutants lacking full length, 144 (ΔC), 86 (ΔC86) and 43 (ΔC43) amino acids of the TMEM174 C-terminal end. C) The bar graph represents the initial FRET ratio of experiments. When NPT2A is partnered with TMEM174wt it exhibits a higher FRET ratio compared to any TMEM174 in which C-terminal end truncations were made. [file NIHPPRS9857363V1-supplement-1.pdf]
